# MicroRNA-218-5p-*Ddx41* axis restrains microglia-mediated neuroinflammation through downregulating type I interferon response in a mouse model of Parkinson’s disease

**DOI:** 10.1186/s12967-024-04881-w

**Published:** 2024-01-16

**Authors:** Danlei Wang, Hongling Gao, Qixiong Qin, Jingyi Li, Jingwei Zhao, Yi Qu, Jiangting Li, Yongjie Xiong, Zhe Min, Zhijuan Mao, Zheng Xue

**Affiliations:** 1 Department of Neurology, Tongji Hospital, Tongji Medical College, Huazhong University of Science and Technology, Wuhan, China; 2 Department of Neurology, The First Affiliated Hospital of Nanchang University, Nanchang, Jiangxi China; 3 Department of General Practice, Tongji Hospital, Tongji Medical College, Huazhong University of Science and Technology, Wuhan, China

**Keywords:** Parkinson’s disease, Microglia, Neuroinflammation, miR-218-5p, Interferon, DEAD-box helicase 41

## Abstract

**Background:**

Parkinson’s disease (PD) is a neurodegenerative disorder characterized by the loss of dopaminergic (DA) neurons in the substantia nigra (SN). Microglia-mediated neuroinflammation has been largely considered one of main factors to the PD pathology. MicroRNA-218-5p (miR-218-5p) is a microRNA that plays a role in neurodevelopment and function, while its potential function in PD and neuroinflammation remains unclear.

**Methods:**

We explore the involvement of miR-218-5p in the PD in a 1-methyl-4-phenyl-1,2,3,6-tetrahydropyridine (MPTP)-induced mouse model. The miR-218-5p agomir used for overexpression was delivered into the substantia nigra (SN) by bilateral stereotaxic infusions. The loss of dopaminergic (DA) neurons and microglial inflammation in the SN was determined using Western blotting and immunofluorescence. Motor function was assessed using the rotarod test. RNA sequencing (RNA-seq) was performed to explore the pathways regulated by miR-218-5p. The target genes of miR-218-5p were predicted using TargetScan and confirmed using dual luciferase reporter assays. The effects of miR-218-5p on microglial inflammation and related pathways were verified in murine microglia-like BV2 cells. To stimulate BV2 cells, SH-SY5Y cells were treated with 1-methyl-4-phenylpyridinium (MPP^+^) and the conditioned media (CM) were collected.

**Results:**

MiR-218-5p expression was reduced in both the SN of MPTP-induced mice and MPP^+^-treated BV2 cells. MiR-218-5p overexpression significantly alleviated MPTP-induced microglial inflammation, loss of DA neurons, and motor dysfunction. RNA sequence and gene set enrichment analysis showed that type I interferon (IFN-I) pathways were upregulated in MPTP-induced mice, while this upregulation was reversed by miR-218-5p overexpression. A luciferase reporter assay verified that *Ddx41* was a target gene of miR-218-5p. In vitro, miR-218-5p overexpression or *Ddx41* knockdown inhibited the IFN-I response and expression of inflammatory cytokines in BV2 cells stimulated with MPP^+^-CM.

**Conclusions:**

MiR-218-5p suppresses microglia-mediated neuroinflammation and preserves DA neurons via *Ddx41*/IFN-I. Hence, miR-218-5p-*Ddx41* is a promising therapeutic target for PD.

**Supplementary Information:**

The online version contains supplementary material available at 10.1186/s12967-024-04881-w.

## Background

Parkinson’s disease (PD) is a major neurodegenerative disease with motor and non-motor symptoms [1, 2], and no cure or effective therapy has been found to delay the disease course. The main pathological feature of PD is the progressive loss of dopaminergic (DA) neurons in the substantia nigra (SN) [3]. Multiple etiologies contribute to the PD pathology, including genetic factors, aging, neurotoxins, insecticides, traumatic brain injury [4], most of which are closely related to the neuroinflammation. As vital innate immune cells of the central nervous system (CNS), microglia are essential participants in neuroinflammation [5]. Imaging, histological and molecular evidence has highlighted microglia-mediated neuroinflammation in PD [6–8]. The degree of microglial activation in PD correlates with dopaminergic degeneration and motor impairment [8], suggesting that the neuroinflammation of resident microglia contributes to the progressive pathogenesis of the disease.

MicroRNAs (miRNAs) are short non-coding RNAs that regulate the transcription and translation process of genes by base-pairing with 3′-untranslated regions (3′UTR) of target genes [9]. Recent evidence has indicated that aberrant miRNA expression drives the development of neuroinflammation in PD [10–13]. MiR-218-5p is an important regulator of neuronal function, whose reduction has been linked to amyotrophic lateral sclerosis [14], cognitive impairment [15] and depression [16]. In PD, miR-218-5p is downregulated in the brain of 6-hydroxydopamine (6-OHDA)-treated rats [17] and patients with PD [18], and its overexpression attenuates survival of dopaminergic neurons by regulating apoptosis and oxidative stress [17]. However, it remains unclear whether miR-218-5p inhibits microglia-mediated neuroinflammation.

Here, we demonstrate that miR-218-5p attenuates microglia-mediated neuroinflammation and preserves DA neurons via *Ddx41*/type I interferon (IFN-I) in a 1-methyl-4-phenyl-1,2,3,6-tetrahydropyridine (MPTP)-induced mouse model. Our results provide a potential therapeutic target for PD, and have important implications for the interpretation of the molecular mechanisms underlying the development of PD and neuroinflammation.

## Methods

### Animals

Eight-week-old male C57BL/6J mice (weighing 19–23 g) were obtained from Gempharmatech (Nanjing, China). Mice were maintained under standard conditions (23 ± 2 °C) on a 12-h light/dark cycle, with ad libitum access to food and water in a specific pathogen-free class facility. All animal experiments were approved by the Tongji Hospital Animal Ethics Committee.

### MPTP treatment

A mouse model of PD was generated using MPTP. Ten mice were randomly assigned to either the control or MPTP group. Mice in the MPTP group were injected intraperitoneally with MPTP·HCl (20 mg/kg free base; M0896, Sigma-Aldrich, St. Louis, MO, USA) in phosphate buffer (PBS) at 2-h intervals for a total of four doses in a single day, while the control group received a similar volume of PBS injected intraperitoneally.

### Stereotaxic injections

We microinjected either miR-218-5p agomir (miR-218) or control agomir (NC) (Ribobio, Guangzhou, China) into the SN using a stereotaxic apparatus to overexpress miR-218-5p. MiRNA agomir is a chemically modified miRNA mimic that has higher stability and activity than conventional miRNA mimics and can effectively simulate the function of endogenous miRNAs. It can be delivered by either systemic or local injection and can exert a lasting effect for up to 6 weeks. Previous studies have demonstrated that miRNA agomirs can effectively regulate gene expression in various tissues such as the brain [19–21]. In this experiment, forty-eight mice were randomly assigned to four groups: NC Control, NC MPTP, miR-218 Control and miR-218 MPTP. Mice were anesthetized with isoflurane (1.5–3% in oxygen) via inhalation and placed in a stereotaxic frame. Then, 0.5 nmol of either miR-218-5p agomir or NC agomir in 2.5 μL of PBS was infused over 10 min into each side of SN at the following coordinates: anteroposterior, − 3.0 mm; mediolateral, ± 1.2 mm; dorsoventral, − 4.7 mm. After each infusion, the needle was left in place for 10 min before being slowly withdrawn. The mice were maintained in a warm environment until they recovered from anesthesia. Mice in the NC Control and miR-218 Control groups were injected with PBS, whereas mice in the NC MPTP and miR-218 MPTP groups were injected with MPTP as described above, 48 h after stereotaxic injection.

### Rotarod test

Motor function was assessed using the rotarod treadmill (IITC, Woodland Hills, CA, USA). Before the formal test, mice underwent three days of training on the rotarod treadmill at a constant speed of 5 rpm for 10 min each day. Thirteen days after the MPTP injection, mice were subjected to the formal test, in which the rod was programmed to accelerate uniformly from 5 to 40 rpm within 5 min. The test was repeated three times for each mouse, and the average latency to fall off the rod was recorded as a measure of motor function. Mice that did not fall off the rod after 5 min were recorded as 300 s.

### Preparation of brain samples

Mice were sacrificed under deep anesthesia induced by isoflurane anesthesia. For immunofluorescence, mice were perfused transcardially with 30 mL of precooled PBS, followed by 20–30 mL of precooled 4% paraformaldehyde (PFA). After perfusion, intact brains were harvested, post-fixed in 4% PFA at 4 °C overnight, and then dehydrated in PBS containing 30% sucrose for 3 days. Brains were embedded in optimal cutting temperature material, followed by cutting into 20-µm thick coronal sections with a freezing slicer (Thermo Fisher Scientific, Waltham, MA, USA). For SN tissue, mice were perfused with 30 mL of precooled PBS, then brains were quickly taken out and continuously sliced at 1 mm thickness in the midbrain coronal plane using a pre-cooled mouse brain matrice (RWD Life Science, Shenzhen, China). SN tissue was segmented under a low magnification microscope according to a mouse brain atlas (The Mouse Brain in Stereotaxic Coordinates, third edition), snap-frozen in liquid nitrogen-cooled isopentane, and stored at − 80 °C.

### Immunofluorescence analysis

The SN slides were fixed with 4% PFA for 10 min and washed three times with PBS for 5 min each time. The tissue area was outlined using a hydrophobic PAP pen. The slices were then blocked with a blocking solution (Beyotime, Shanghai, China) and incubated with primary antibodies overnight at 4 °C. Subsequently, the slices were incubated with secondary antibodies at room temperature in the dark for 1 h. After being washed with PBS, the slices were mounted with The Antifade Mounting Medium with DAPI (Beyotime) and covered with coverslips. The primary antibodies used for immunofluorescence were as follows: anti-tyrosine hydroxylase (TH; rabbit, 1:500; ab137869, Abcam, Cambridge, UK), anti-IBA1 (rabbit, 1:500; 019-19741, Fujifilm Wako Chemical, Osaka, Japan), anti-CD68 (rat, 1:500; MCA341B, Bio-Rad, Hercules, CA) and anti-IRF7 (mouse, 1:200; sc-74471, Santa Cruz Biotechnology, Dallas, TX, USA). The secondary antibodies were labeled with Alexa Fluor 488 or 594 (1:400, Yeasen Biotechnology, Shanghai, China). Confocal microscopy (FV1200; Olympus, Tokyo, Japan) was used to acquire images. To quantify TH^+^ neurons in the SNc, we employed a stereological approach, as previously described [22]. Slices from another 8 sections (140-μm interval) per mouse were collected, and 10 z-stacks (2 μm per stack) confocal images at 10 × magnification were acquired for neuron counting using Fiji 2.9.0 (ImageJ; National Institutes of Health, Bethesda, MD, USA). To quantify IBA1^+^ cells and volumes of CD68^+^IBA1^+^ puncta, images were obtained at 60 × magnification with 10 z-stacks (2-μm per stack). We randomly selected three fields and analyzed three sections per mouse. We performed 3D reconstruction of IBA1^+^ cells and CD68^+^ puncta using the Surface module in Imaris 9.0.3 (Oxford Instrument, Belfast, UK). The volume of IBA1^+^ cells and CD68^+^ puncta was calculated and averaged from 10 to 15 IBA1^+^ cells randomly selected from each mouse. To quantify the average fluorescent intensity of IRF7 + IBA1 + cells, images were captured at 60 × magnification with 6 z-stacks, and three fields were taken at random in each section, and three sections per mouse were analyzed. Microglia were selected by setting a fixed threshold in the IBA1 channel in Fiji, and then the average fluorescent intensity of IRF7 in microglia was calculated.

### RNA sequencing and bioinformatics analysis

RNA sequencing (RNA-seq) was performed on SN tissues from mice in the NC control group, NC MPTP group, miR-218 control group, and miR-218 MPTP group (three each). RNA extraction, library construction, sequencing and bioinformatics analysis were performed by Suzhou PANOMIX Biomedical Tech Co. (Suzhou, China). The specific process involved the following steps: firstly, polyA-tailed mRNA was enriched from total RNA using oligo (dT) magnetic beads, and the RNA was then ion-fragmented to produce fragments of around 300 bp. Reverse transcription was performed using RNA as a template, 6-mer random primers and reverse transcriptase to synthesize first-strand cDNA, which was subsequently used to synthesize the second-strand cDNA. After library construction, PCR amplification was used to enrich the library fragments, which were then selected based on size to obtain a final library size of 450 bp. The quality of the library was assessed using the Agilent 2100 Bioanalyzer (Santa Clara, CA, USA), and the total and effective concentrations of the library were determined. The libraries with different index sequences were mixed in proportion to one another based on the effective concentration of the library and the required data amount. The mixed library was diluted to 2 nM and converted to single-stranded DNA using alkaline denaturation. Next-generation sequencing (NGS) was performed on the Illumina sequencing platform (San Diego, CA, USA) using paired-end (PE) sequencing. The libraries were sequenced to a depth of ~ 6 Gbps per sample. The resulting images were converted into raw data in FASTQ format using the sequencing platform's proprietary software. The sequencing data was filtered according to two criteria: (1) removal of 3′ adapter sequences using Cutadapt, and (2) removal of reads with an average quality score below Q20. The resulting high-quality sequences were mapped to the reference genome (Mus musculus.GRCm38.dna.primary_assembly.fa). HTSeq was used to count the number of reads mapped to each gene, which was used as the gene's raw expression level. The expression levels were then normalized using Fragments Per Kilo bases per Million fragments (FPKM).

We performed principal component analysis (PCA) using the DESeq package in R on each sample based on gene expression levels. The gene expression analysis was conducted using the DESeq package in R, and differentially expressed genes were screened with criteria of |log2FoldChange|> 0.585 and *p*-value < 0.05. Gene set enrichment analysis (GSEA) was conducted using a local GSEA tool (http://www.broadinstitute.org/gsea/index.jsp) and the Gene Ontology (GO) data set of mice. The normalized enrichment score (NES) and false discovery rate (FDR) q-value were calculated using permutation tests to quantify enrichment levels and statistical significance. Pathways with significant enrichment were defined as those meeting the following criteria: |NES|> 1, the nominal (NOM) *p*-value < 0.05, and FDR q-value < 0.25.

### Cell culture and treatment

Murine microglia-like BV2 cells, SH-SY5Y cells and human embryonic kidney (HEK) 293 T cells were maintained in high-glucose Dulbecco’s modified Eagle’s medium (DMEM; Gibco, Thermo Fisher Scientific) containing 10% fetal bovine serum (FBS; Biological Industries, Beit-Haemek, Israel) in a carbon dioxide incubator at 37 °C. The cells were subcultured every three days. For 1-methyl-4-phenylpyridinium (MPP^+^; D048, Sigma-Aldrich) treatment, SH-SY5Y cells were treated with 1 mM MPP^+^ or PBS of equal volume for 24 h. The conditioned media from the SH-SY5Y cells treated with MPP^+^ (MPP^+^-CM) or PBS (PBS-CM) were collected and applied to BV2 cells for 24 h.

### Cell transfection

BV2 cells were digested and plated into 6-well plates in a high-glucose DMEM medium containing 10% FBS one day before transfection. Transfection was performed when the cells reached ~ 80% confluence. BV2 cells were transfected with miR-218-5p mimic/NC mimic or *Ddx41*-siRNA/control siRNA (Ribobio) for 6–8 h with LipofectMax transfection reagent (ABP Biosciences, Beltsville, MD, USA), and then treated with MPP^+^-CM or PBS-CM for 24 h.

### Western blot (WB) analysis

Samples from SN tissue and BV2 cells were lysed using RIPA lysis buffer (Beyotime) containing a protease inhibitor cocktail and phenylmethylsulfonyl fluoride. Protein concentrations were determined using a bicinchoninic acid kit (Beyotime). Protein content (20 μg) was loaded onto sodium dodecyl sulfate–polyacrylamide gel for gel electrophoresis and transferred to nitrocellulose membranes, which were subsequently blocked with 5% non-fat skim milk and incubated with the primary antibodies at 4 °C overnight. The primary antibody used for WB were as follows: anti-TH (rabbit, 1:1000; ab137869, Abcam), anti-DDX41 (rabbit, 1:1000; 27,500–1-AP, Proteintech, Rosemont, IL, USA), anti-IL-1β (rabbit, 1:1000; #12703, Cell Signaling Technology, Danvers, MA, USA), anti-GAPDH (mouse, 1:10,000; 60,004-1-Ig, Proteintech) and anti-β-actin (mouse, 1:10,000; 66,009-1-Ig, Proteintech). Secondary antibody reactions were performed using goat anti-mouse or goat anti-rabbit IgG-horseradish peroxidase antibodies (1:5000; Proteintech) for 1 h at room temperature. WB protein bands was visualized by enhanced chemiluminescence. Images were collected using the BLT GelView 6000 Pro imaging system (Guangzhou Biolight Biotechnology, Guangzhou, China). To quantify WB protein bands, equally sized region of interest was drawn around each band in Fiji to measure raw integrated density. Each integrated density was normalized to each sample’s control (GAPDH or β-actin).

### Quantitative real-time PCR (qPCR) analysis

Total RNA was extracted from the SN tissue of mice and cells using RNAiso Plus reagent (Takara, Kusatsu, Japan). RNA reverse transcription and qPCR amplification were performed using PrimeScript RT Reagent Kits (RR036A and RR037A; Takara) and ChamQ Universal SYBR qPCR Master Mix (Vazyme, Nanjing, China), respectively. The primers for miR-218-5p and U6 were obtained from the Bulge-Loop miRNA qPCR Primer Set (Ribobio). The primers for mRNA provided by Tsingke Biotechnology (Beijing, China) are listed in Additional file 1: Table S1. qPCR amplifications were performed using CFX Connect Detection System (Bio-Rad) as follows: 3 min at 95 °C, 10 s at 95 °C for 40 cycles, 30 s at 55 °C. qPCR was performed using the 2 ^−ΔΔCt^ method with *Actb* (for mRNA) or *U6* (for miR-218-5p) as controls.

### Dual luciferase reporter analysis

HEK 293 T cells were seeded one day before transfection. miR-218-5p mimic or NC mimic were co-transfected with pmirGLO vectors, including wild-type (WT) or mutated (MUT) 3′UTR of *Ddx41*. Luciferase activity was measured 48 h after transfection, according to the manufacturer’s protocol (GeneCopoeia, Rockville, MD).

### Statistical analysis

GraphPad Prism (v8.0.2; GraphPad Software, San Diego, CA) was applied for the statistical analysis. Two-group comparisons were performed using a two-tailed unpaired Student’s t-test. For multi-group comparisons, two-way analysis of variance (ANOVA) and Tukey’s multiple comparison test were used. Statistical significance was established at *p* < 0.05.

## Results

### miR-218-5p overexpression attenuates PD-associated phenotypes in MPTP-induced mice

**Fig. 1:**
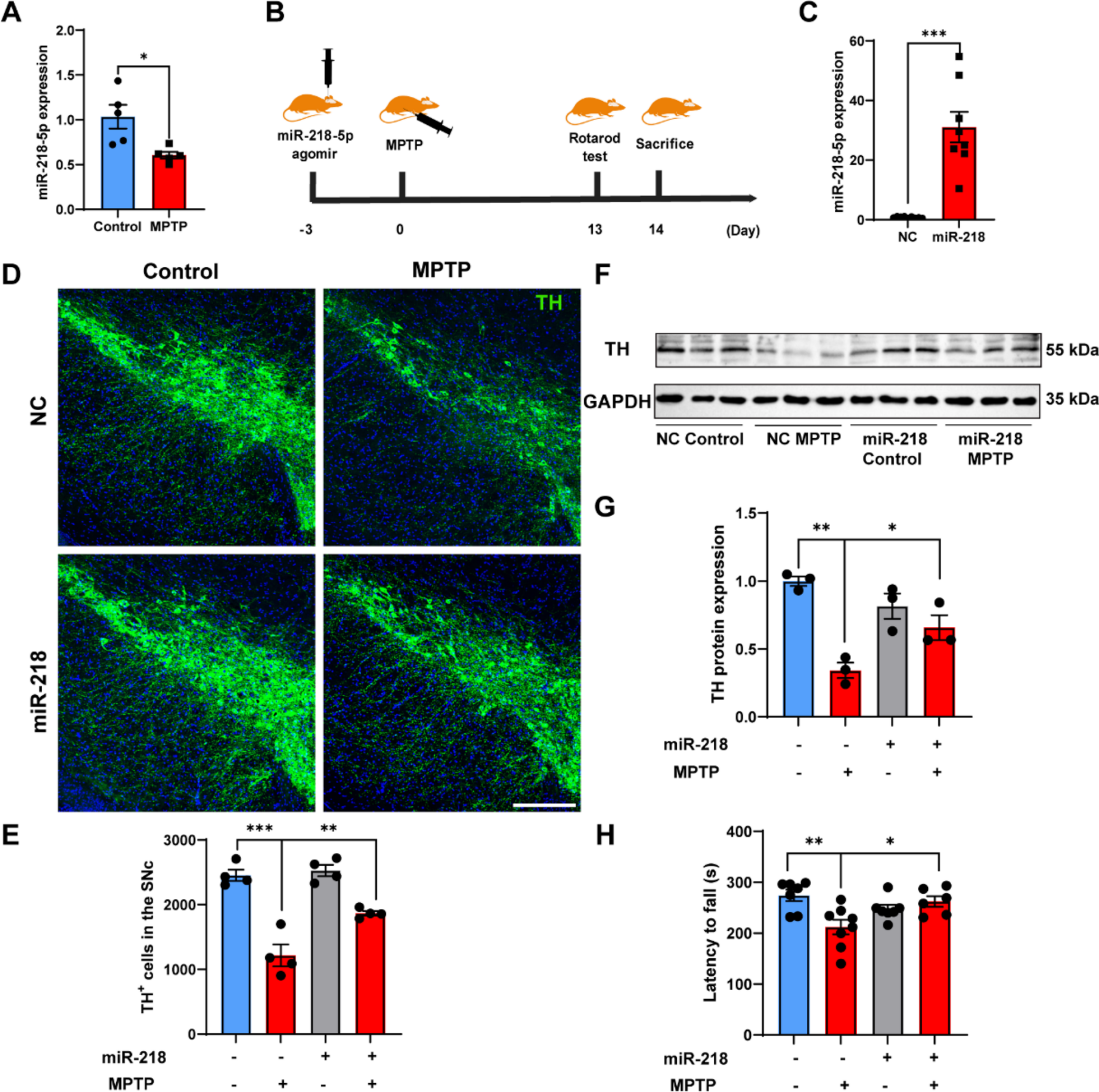
miR-218-5p overexpression alleviates the loss of dopaminergic neurons and motor deficits in MPTP-induced mice. **A** miR-218-5p expression in the SN of Control and MPTP mice 14 days post MPTP administration is shown by qPCR analysis. *N* = 5 per group. **B** A schematic diagram of constructing a miR-218-5p-overexpressing-MPTP mice model. **C** Effective miR-218-5p overexpression in the SN of mice 17 days post miR-218-5p agomir injection was verified by qPCR analysis. miR-218, mice injected with miR-218-5p agomir. NC, mice injected with NC agomir. *N* = 8–9 per group. **D**, **E** Representative confocal images staining of TH in the SN (**D**) and quantification of TH^+^ cells (**E**) of NC Control, NC MPTP, miR-218 Control and miR-218 MPTP mice. Scale bar, 250 μm. *N* = 3–4 per group. **F**, **G** TH expression in the SN by western blot (**F**) and quantitative analysis (**G**) of NC Control, NC MPTP, miR-218 Control and miR-218 MPTP mice. *N* = 3 per group. **H** Quantification of latency to fall in the accelerating Rotarod test of NC Control, NC MPTP, miR-218 Control and miR-218 MPTP mice 13 days post MPTP administration. *N* = 6–8 per group. Data are shown as the mean ± SEM. Significance in **A**, **C** was tested by two-tailed unpaired Student’s t-test. Significance in **E**–**H** was tested by two-way ANOVA. *p < 0.05, **p < 0.01, ***p < 0.001

To evaluate the effect of miR-218-5p on the loss of DA neurons in MPTP-induced mice, we first examined miR-218-5p levels in the SN using qPCR. We found that miR-218-5p expression was downregulated 14 days post MPTP administration (Fig. 1a). Next, mice were stereotaxically infused with miR-218-5p agomir (or NC agomir) into the SN and then injected intraperitoneally with MPTP 3 days after stereotaxic injection (Fig. 1b). miR-218-5p agomir treatment significantly increased miR-218-5p expression after 17 days (Fig. 1c). The number of TH^+^ DA neurons and TH expression were reduced in MPTP- and NC agomir-treated mice, as determined by immunofluorescence and WB analyses. However, miR-218-5p agomir treatment alleviated the reduction in TH^+^ neurons and TH expression (Fig. 1d–g). Moreover, miR-218-5p overexpression significantly attenuated the MPTP-induced behavioral deficits in the rotarod test 13 days post MPTP injection (Fig. 1h). These data indicate that miR-218-5p overexpression alleviates the loss of DA neurons and motor deficits in MPTP-induced mice.

### miR-218-5p overexpression inhibits microglia-mediated neuroinflammation in the SN of MPTP-induced mice

**Fig. 2:**
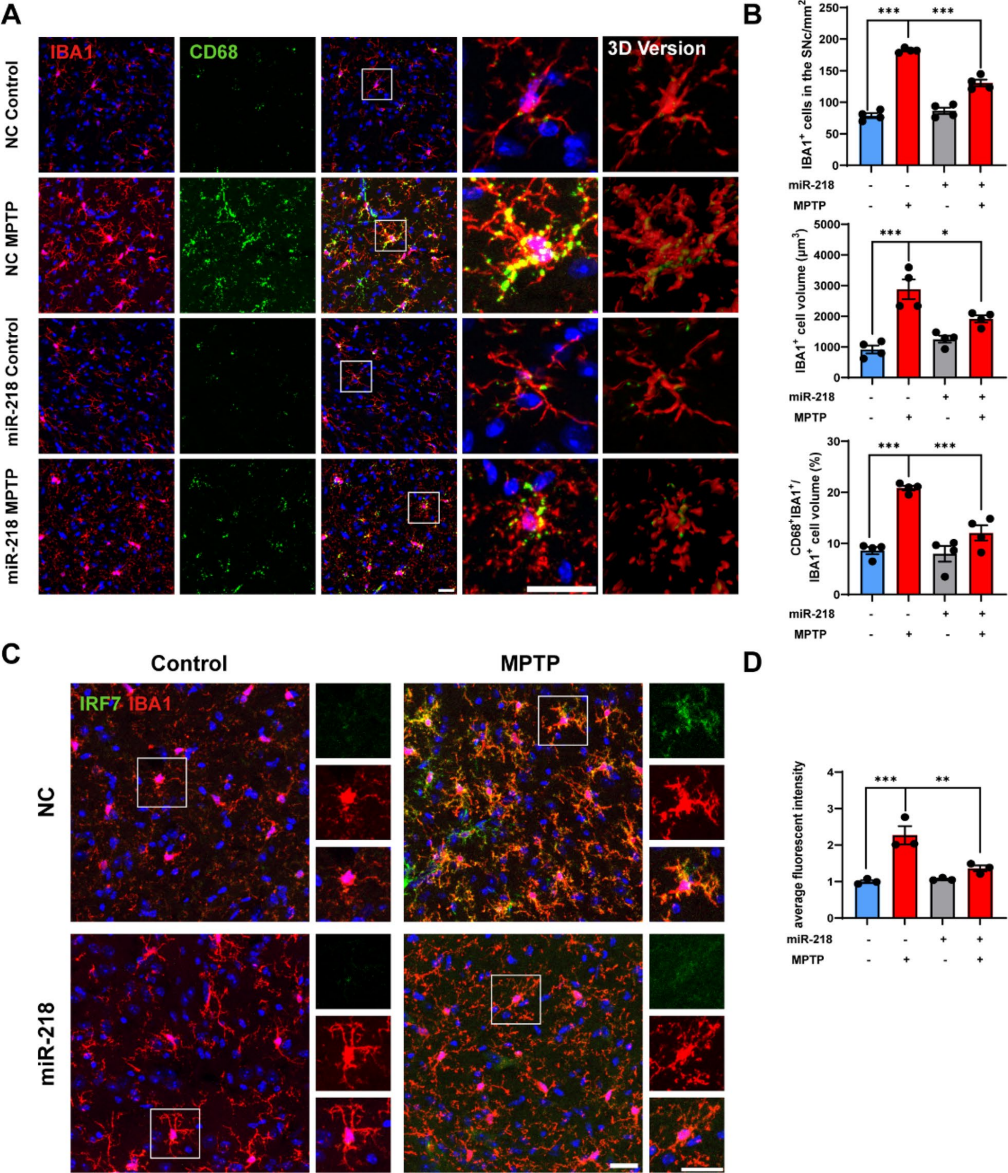
miR-218-5p overexpression inhibits microglial inflammatory and IFN-I responses in the SN in MPTP-induced mice. **A** Representative confocal images and 3D reconstructed images staining of IBA1 (red) and CD68 (green) in the SN of NC Control, NC MPTP, miR-218 Control and miR-218 MPTP mice. Scale bar, 25 μm.** B** Quantification of IBA1^+^ cells, IBA1^+^ cell volume and the ratio of CD68^+^IBA1^+^ puncta volume to IBA1^+^ cell volume in the SN of NC Control, NC MPTP, miR-218 Control and miR-218 MPTP mice. *N* = 4 per group. **C** Representative confocal images staining of IBA1 (red) and IRF7 (green) in the SN of NC Control, NC MPTP, miR-218 Control and miR-218 MPTP mice. Scale bar, 25 μm. **D** Quantification of the average intensity of IRF7 protein in IBA1^+^ cells in the SN of NC Control, NC MPTP, miR-218 Control and miR-218 MPTP mice. Data are shown as the mean ± SEM. Significance was tested by two-way ANOVA. *p < 0.05, **p < 0.01, ***p < 0.001

To explore whether miR-218-5p overexpression affects microglial phenotypes in MPTP-induced mice, double immunofluorescence staining with anti-IBA1 (a marker for microglia) and anti-CD68 (a marker for phagocytosis) was performed to identify microglial inflammation in the SN (Fig. 2a). The number and volume of IBA1^+^ microglia, as well as CD68 puncta in IBA1^+^ microglia were significantly increased in the SN of MPTP-induced mice, whereas these effects were attenuated in mice with miR-218-5p overexpression (Fig. 2a, b). Additionally, MPTP administration increased the level of pro-inflammatory cytokine IL-1β, whereas overexpression of miR-218-5p inhibited it (Fig. 5b, c). This suggests that miR-218-5p overexpression inhibits MPTP-induced microglia-mediated neuroinflammation.

### miR-218-5p overexpression inhibits the IFN-I response in microglia of the SN of MPTP-induced mice

**Fig. 3:**
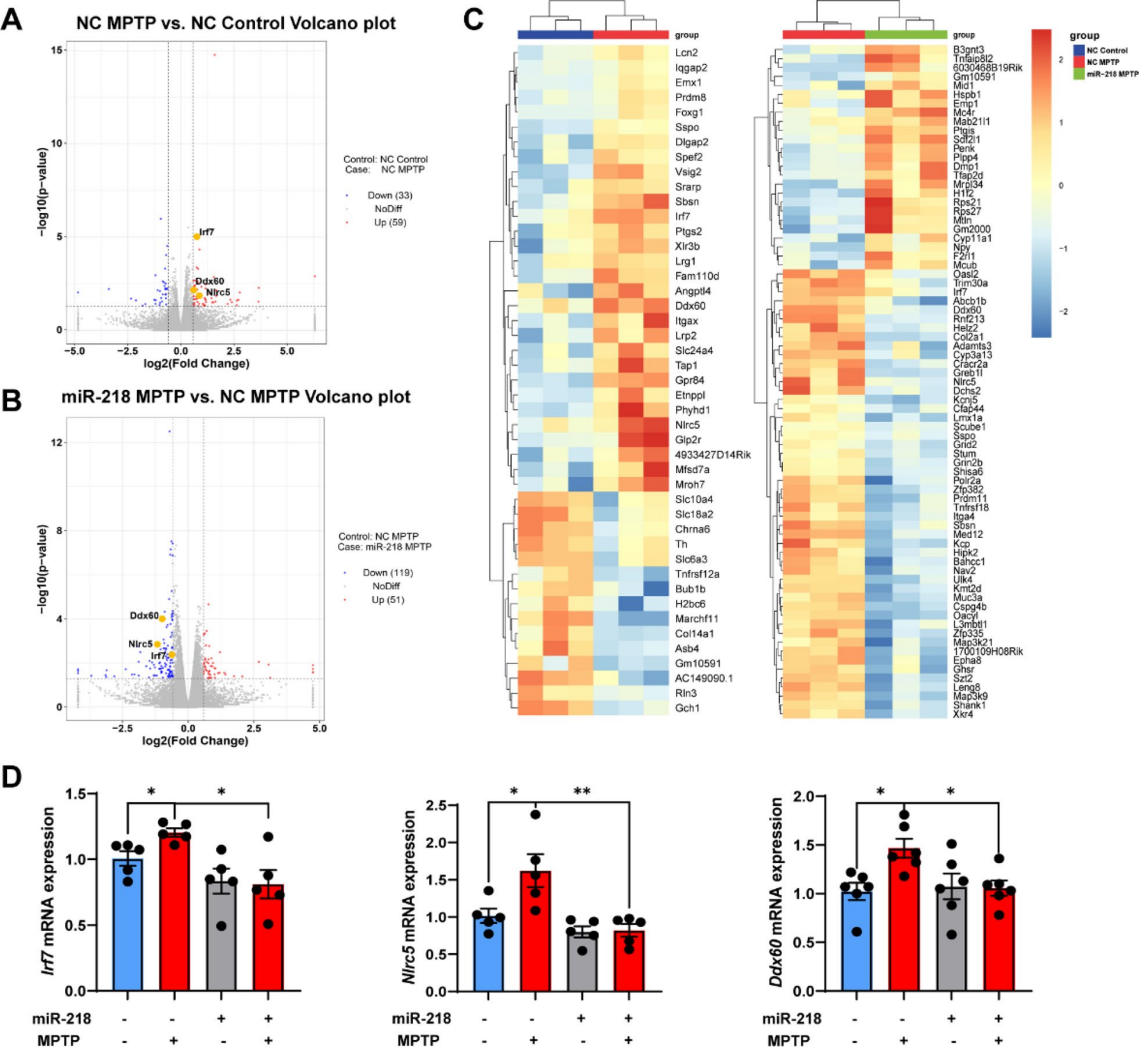
miR-218-5p overexpression inhibits the expression of IFN-I response related genes in the SN of MPTP-induced mice. **A**, **B** Volcano plots showing the DEGs in NC MPTP mice versus NC Control mice (**A**) and the DEGs in miR-218 MPTP mice versus NC MPTP mice (**B**). *N* = 3 per group. Data are shown as |Log2 (fold change)|≥ 0.585, *p*-value < 0.05. **C** Pheatmaps showing the top DEGs in NC MPTP mice versus NC Control mice and the top DEGs in miR-218 MPTP mice versus miR-218 Control mice. **D** qPCR analysis showing mRNA expression of several IFN-I related genes (*Irf7*, *Nlrc5*, *Ddx60*) in the SN of NC Control, NC MPTP, miR-218 Control and miR-218 MPTP mice. *N* = 5–6 per group. Data are shown as the mean ± SEM. Significance was tested by two-way ANOVA. *p < 0.05, **p < 0.01

**Fig. 4:**
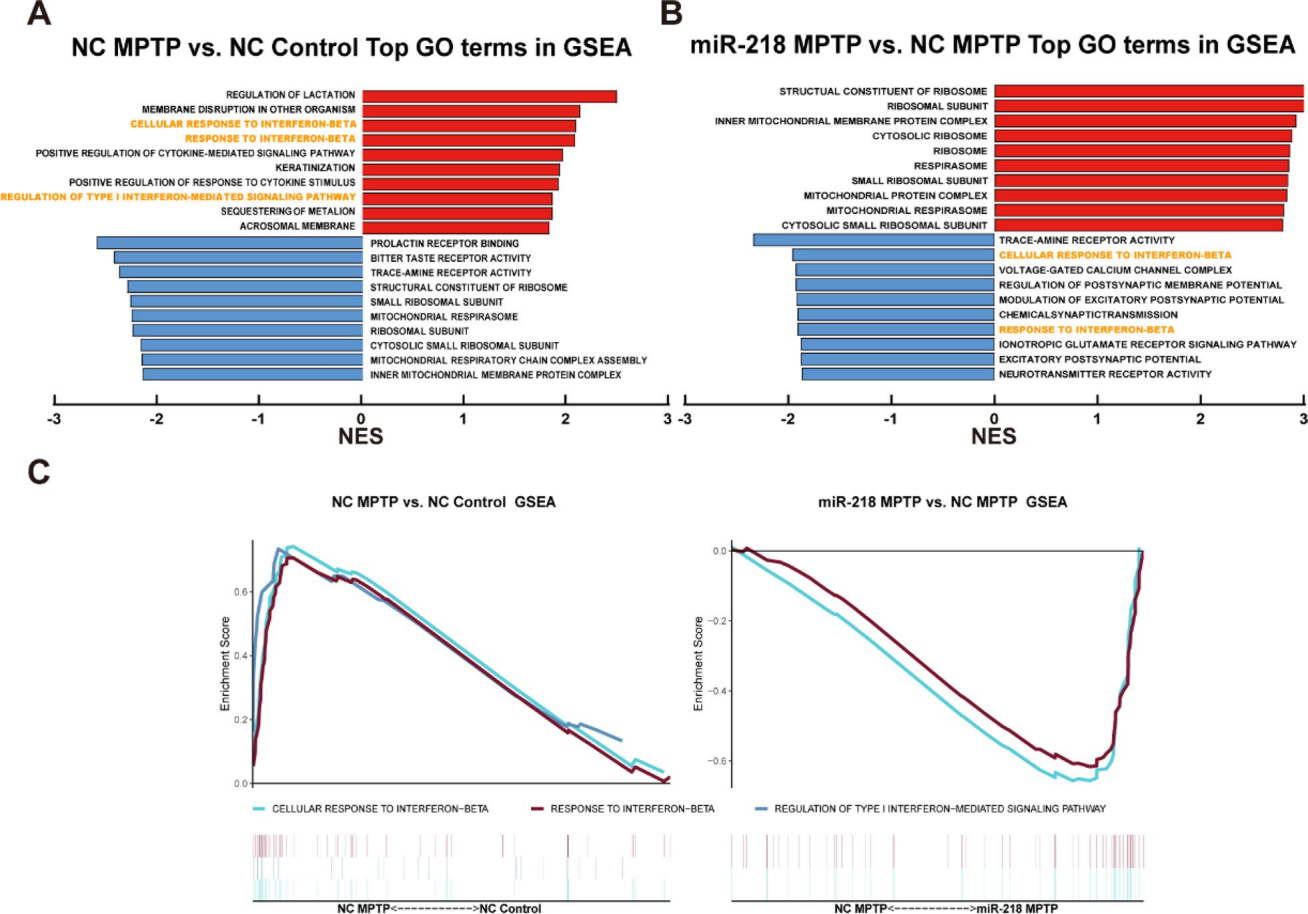
Top GO enrichment terms in GSEA of NC Control, NC MPTP, miR-218 Control and miR-218 MPTP mice. **A** Top GO enrichment terms are shown in GSEA of NC MPTP mice versus NC Control mice. **B** Top GO enrichment terms are shown in GSEA of miR-218 MPTP mice versus NC MPTP mice. **C** Several IFN-I responses terms are shown in GSEA of NC MPTP mice versus NC Control mice and miR-218 MPTP mice versus NC MPTP mice. |NES|> 1, NOM *p*-value < 0.05, and FDR q-value < 0.25 are considered to be of interest

To determine the mechanism by which miR-218-5p affects microglial inflammation, we performed RNA-seq on the SN tissue of mice. Volcano plots of DEG analysis showed that 59 genes were upregulated and 33 genes were downregulated in the SN of MPTP-induced mice (Fig. 3a). However, miR-218-5p overexpression upregulated 119 genes and downregulated 51 genes in MPTP-induced mice (Fig. 3b). Notably, the expression of several IFN-I-related genes (*Irf7*, *Ddx60*, *Nlrc5*) was upregulated in MPTP-treated mice (Fig. 3a, c), while miR-218-5p overexpression downregulated the expression of these genes (*Irf7*, *Ddx60*, *Nlrc5*) (Fig. 3b, c). We verified the expression of *Irf7*, *Ddx60*, and *Nlrc5* by qPCR (Fig. 3d). Moreover, miR-218-5p overexpression led to decreased expression of several genes associated with IFN-I and inflammatory responses in the baseline (Additional file 1: Fig. S1). The top differential GO terms with *p*-value in GSEA are shown in Fig. 4a, b. We found that MPTP treatment upregulated several IFN-I response-related terms, including “cellular response to interferon-beta”, “response to interferon-beta, and “regulation of type I interferon-mediated signaling pathway” (Fig. 4a, c; Additional file 1: Fig. S2). Yet, miR-218 overexpression downregulated IFN-I terms including “cellular response to interferon-beta” and “response to interferon-beta” (Fig. 4b, c; Additional file 1: Fig. S2). Our data suggest that the IFN-I response is upregulated in the MPTP mouse model of PD, which may be due to the downregulation of miR-218-5p.

To investigate if miR-218-5p inhibits IFN-I response in microglia, we performed double immunofluorescence staining with anti-IBA1 and anti-IRF7 antibodies in the SN (Fig. 2c). The IRF7 protein was predominantly distributed in IBA1^+^ microglia (Fig. 2c), and the fluorescent intensity of IRF7 in microglia was elevated in MPTP-induced mice, while miR-218-5p overexpression suppressed its expression (Fig. 2d). This suggests that miR-218-5p regulates the IFN-I response of microglia and may modulate neuroinflammation through this pathway.

### miR-218-5p targets *Ddx41*, an IFN-I-related gene

**Fig. 5:**
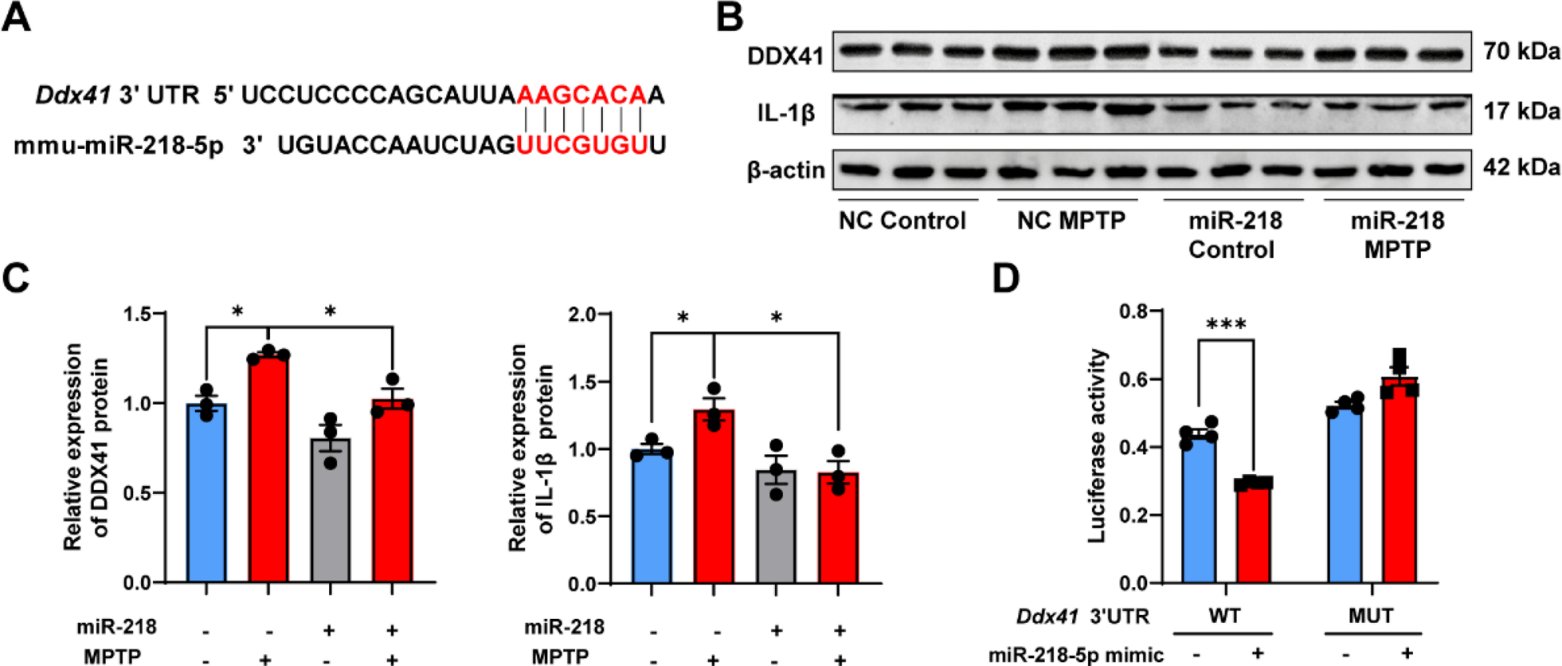
*Ddx41* is a target gene of miR-218-5p. **A** The binding sites of mmu-miR-218-5p with *Ddx41* 3′UTR. **B**, **C** DDX41 expression in the SN by western blot (**B**) and quantitative analysis (**C**) of NC Control, NC MPTP, miR-218 Control and miR-218 MPTP mice. *N* = 3 per group. **D** PmirGLO vectors including wildtype (WT) or mutated (MUT) 3′UTR of *Ddx41* were co-transfected with miR-218-5p mimic or mimic NC in HEK293T cells. Luciferase activity was measured. *N* = 4 per group. Data are shown as the mean ± SEM. Significance was tested by two-way ANOVA. *p < 0.05, **p < 0.01, ***p < 0.001

We next investigated the possible mechanisms of miR-218-5p regulating IFN-I response. TargetScan 7.2 was used to predict target genes of miR-218-5p. We found that the 3′UTR of the IFN-I-related gene *Ddx41* (encoding DEAD-box helicase 41 [DDX41]) is complementary to miR-218-5p (Fig. 5a). DDX41 is a DNA sensor that recognizes viral DNA [23] and cytosolic DNA [24] in the cytoplasm, and subsequently induces IFN-I responses by interacting with STING [25, 26]. DDX41 protein levels were increased in the SN of MPTP-injected mice, as analyzed by WB analysis, while DDX41 expression was reduced following miR-218-5p overexpression (Fig. 5b, c). Dual luciferase reporter assays confirmed that miR-218-5p targets *Ddx41* 3′UTR (Fig. 5d). These results suggest that miR-218-5p targets *Ddx41*.

### miR-218-5p overexpression suppresses IFN-I and inflammatory responses in BV2 cells treated with MPP^+^ conditioned media

**Fig. 6:**
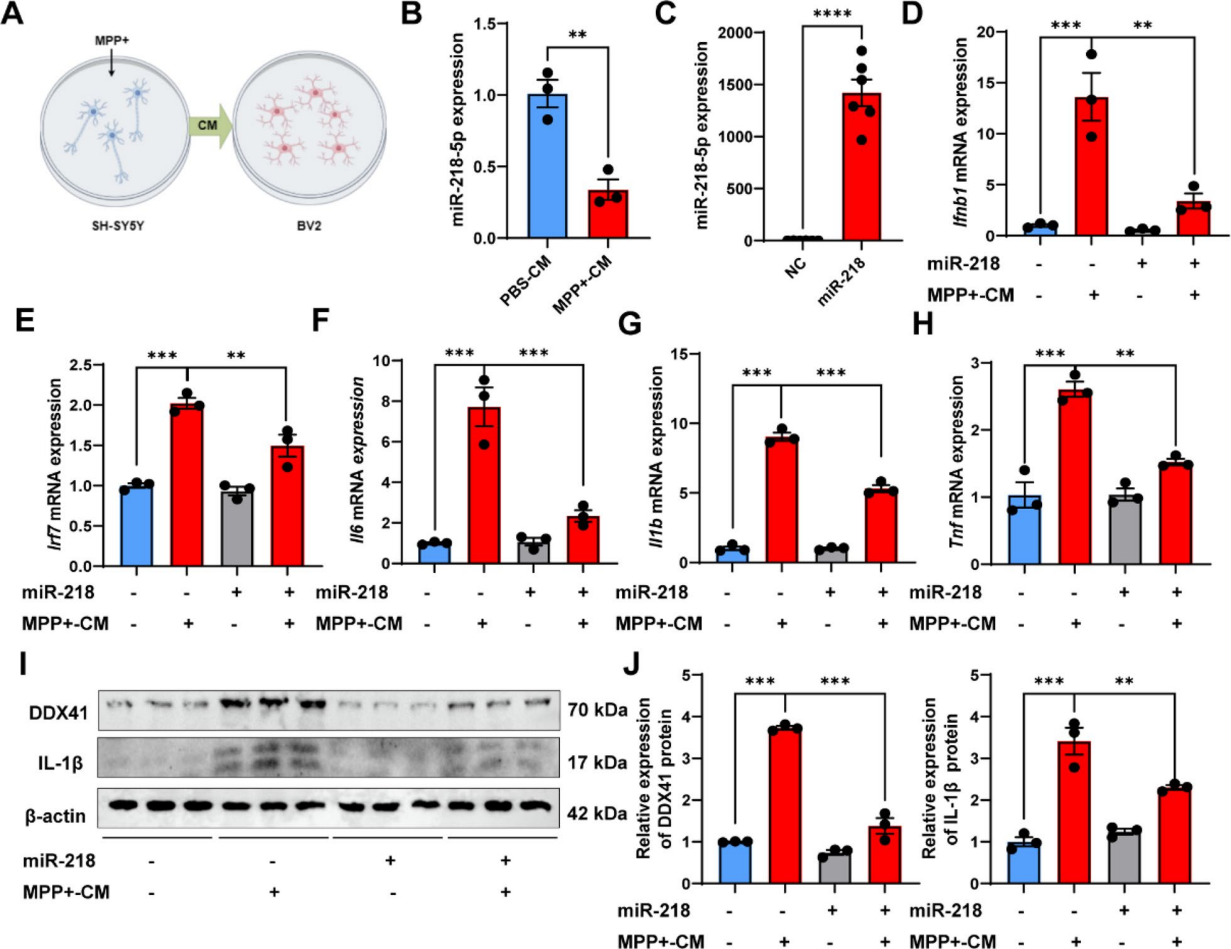
miR-218-5p overexpression suppresses IFN-I and inflammatory responses in BV2 cells treated with MPP + conditioned media. **A** A schematic diagram showing treatment of conditioned medium (CM) from MPP^+^ stimulated SH-SY5Y cells into BV2 cells. **B** miR-218-5p expression in BV2 cells treated with PBS-conditioned media (PBS-CM) and MPP^+^-conditioned media (MPP^+^-CM) is shown by qPCR analysis. *N* = 3 per group. **C** Effective miR-218-5p overexpression in the BV2 cells transfected with miR-218-5p mimic was verified by qPCR analysis. N = 6 per group. **D**–**H** *Ifnb1* (**D**), *Irf7* (**E**), *Il6* (**F**), *Il1b* (**G**) and *Tnf* (**H**) mRNA expression in BV2 cells transfected with miR-218-5p mimic and stimulated with or without MPP^+^-CM is shown by qPCR analysis. *N* = 3 per group. **I**, **J** DDX41 and IL-1β protein levels in BV2 cells transfected with miR-218-5p mimic and stimulated with or without MPP^+^-CM by western blot (**I**) and quantitative analysis (**J**) are shown. *N* = 3 per group. Data are shown as the mean ± SEM, *p < 0.05, **p < 0.01, ***p < 0.001,****p < 0.0001

To elucidate whether miR-218-5p regulates the microglial IFN-I response and affects microglia-mediated inflammation, we established SH-SY5Y-BV2 conditioned culture systems (Fig. 6a), due to the fact that previous studies have suggested that the inflammation of microglia caused by MPTP is secondary to the MPP^+^ induced neurotoxicity [27]. We treated BV2 cells with MPP^+^ conditioned media (MPP^+^-CM) from SH-SY5Y cells, and found that miR-218-5p expression was downregulated in BV2 cells treated with MPP^+^-CM (Fig. 6b). Furthermore, the expression of IFN-I response related genes *Ifnb1* and *Irf7* (Fig. 6d, e) and the protein level of DDX41 (Fig. 6i, j) were upregulated in BV2 cells after MPP^+^-CM stimulation. In contrast, miR-218-5p overexpression (Fig. 6b) downregulated *Ifnb1* and *Irf7* expression (Fig. 6d, e) and DDX41 level (Fig. 6i, j) induced by MPP^+^-CM. Consistent with this, MPP^+^-CM stimulation upregulated the expression of pro-inflammatory cytokines *Il1b*, *Il6* and *Tnf*, whereas miR-218-5p overexpression attenuated their expression (Fig. 6f–h).

### ***Ddx41*** knockdown suppresses IFN-I and inflammatory responses in BV2 cells treated with MPP^+^ conditioned media

**Fig. 7:**
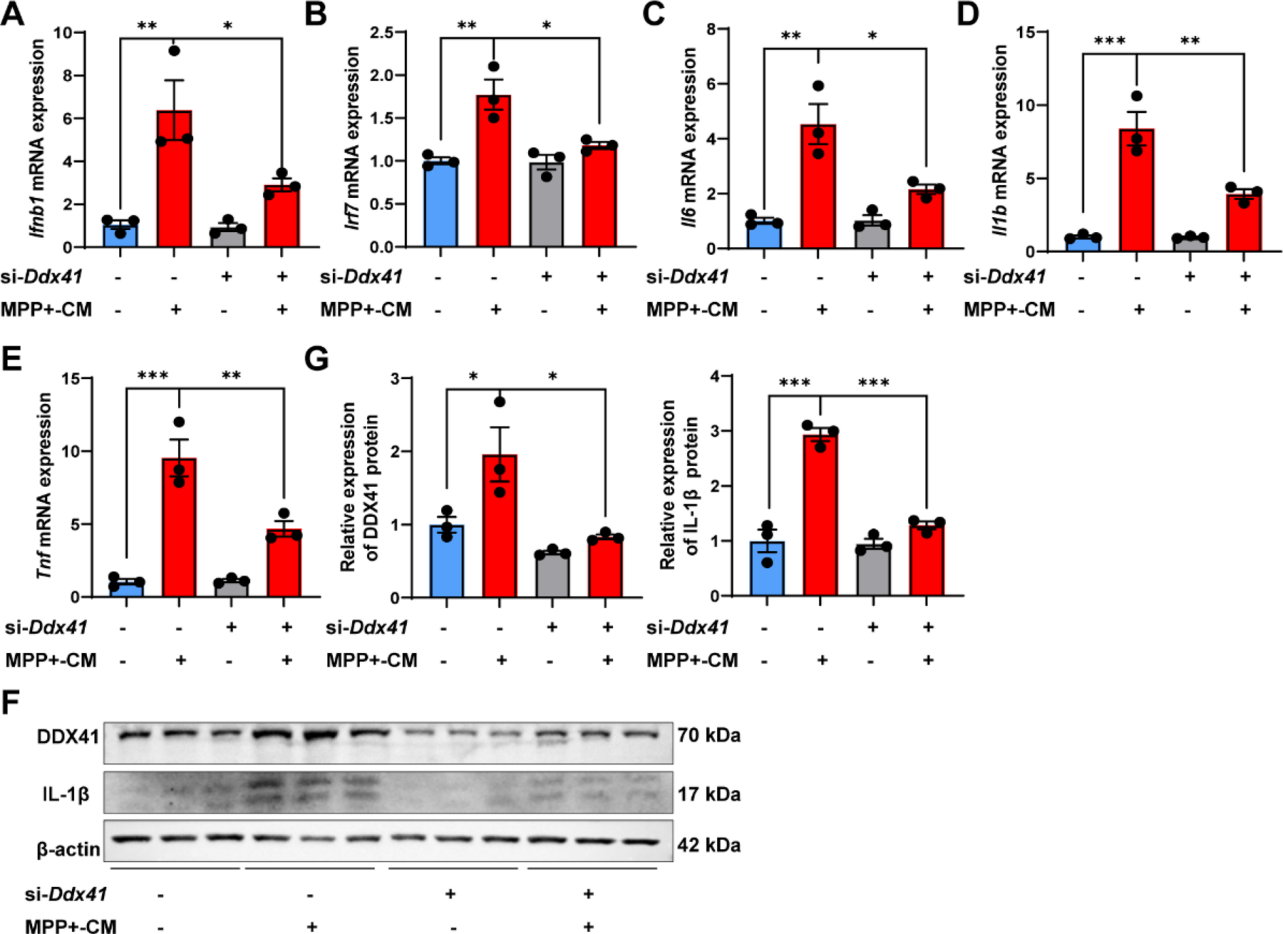
*Ddx41* knockdown suppresses IFN-I and inflammatory responses in BV2 cells treated with MPP^+^ conditioned media. **A**–**E** *Ifnb1* (**A**), *Irf7* (**B**), *Il6* (**C**), *Il1b* (**D**) and *Tnf* (**E**) mRNA expression in BV2 cells transfected with *Ddx41* siRNA and stimulated with or without MPP^+^-CM is shown by qPCR analysis. *N* = 3 per group. **F**, **G** DDX41 and IL-1β protein levels in BV2 cells transfected with *Ddx41* siRNA and stimulated with or without MPP^+^-CM by western blot (**F**) and quantitative analysis (**G**) are shown. *N* = 3 per group. Data are shown as the mean ± SEM, *p < 0.05, **p < 0.01, ***p < 0.001

Next, we investigated whether miR-218-5p regulates microglial IFN-I response and inflammation by inhibiting the expression of *Ddx41*. BV2 cells were transfected with *Ddx41* siRNA (si-*Ddx41*) to knock down *Ddx41*. The expression of *Ifnb1* and *Irf7* and the level of DDX41 were increased in BV2 cells after MPP^+^-CM stimulation, while *Ddx41* knockdown suppressed their expression (Fig. 7a, b, f, g). In addition, the expression of *Il6*, *Il1b* and *Tnf* was elevated in BV2 cells induced by MPP^+^-CM, while *Ddx41* knockdown prevented the elevation of these pro-inflammatory cytokines (Fig. 7c–g).

## Discussion

In the present study, we demonstrated that miR-218-5p was significantly downregulated in the SN of MPTP-induced mouse model of PD. Intriguingly, miR-218-5p overexpression inhibited microglial inflammation and loss of DA neurons in the SN, and alleviated motor deficits in the mouse model. Mechanistically, miR-218-5p inhibited microglia IFN-I responses and inflammation by targeting *Ddx41*. Hence, miR-218-5p-*Ddx41* is a promising therapeutic target for PD.

MiRNAs play important roles in the pathogenesis of PD [28–30]. Previous studies have suggested that miR-218-5p is involved in the development of PD. Our findings agree with previous studies that show miR-218-5p is reduced in 6-OHDA-induced rat models [17] and patients [18], and protects DA neurons from 6-OHDA-induced damage [17]. However, another study showed that miR-218-5p is upregulated in the midbrain of patients with advanced PD [31], suggesting that the expression of miR-218-5p in different stages or brain regions of PD requires further study. Our findings that miR-218-5p was decreased in MPTP-injected mice, and miR-218-5p overexpression mitigated the loss of DA neurons in these mice, support that miR-218-5p protects against neurodegeneration in PD.

MiR-218-5p was implicated in neuronal differentiation [32–34] and tumor suppression [35–37]. Recent research work has revealed that miR-218-5p can also regulate inflammatory processes. In the periphery, bronchial epithelial miR-218-5p inhibits airway inflammation in asthma [38] and chronic obstructive pulmonary disease [39]. MiR-218-5p also targets IKK-β to regulate NK-κB-mediated inflammation in diabetic nephropathy [40]. In the CNS, miR-218-5p inhibits neuroinflammation in diabetic encephalopathy via targeting TLR4. Moreover, the level of miR-218-5p is negatively correlated with the levels of pro-inflammatory cytokines such as TNF-α, IL-1β and IFN-γ in the prefrontal cortex of patients with PD [18]. In line with these reports of the protective role of miR-218-5p in inflammation, we demonstrated that the overexpression of miR-218-5p can reduce the levels of pro-inflammatory cytokines (IL-1β and IL-6) in MPP^+^-treated BV2 cells and alleviate MPTP-induced neuroinflammation in microglia in vivo. It is reported that microglia-mediated neuroinflammation is an early event in PD and contributes to neuron degeneration [41], suggesting that neuroprotective effects of miR-218-5p may be achieved by inhibiting microglial inflammation.

To further explore how miR-218-5p suppresses neuroinflammation in microglia, we conducted RNA-seq analysis. We found that while the IFN-I response-related pathways were activated in mice treated with MPTP, miR-218-5p overexpression was able to inhibit these pathways. IFNs were initially discovered as antiviral substances [42] and are classified into three types. IFN-I includes IFN-α (encoded by more than ten genes), IFN-β (encoded by a single gene), and several other IFNs [43]. IFN-I responses are primarily mediated by microglia and regulate microglia functions in various neurological diseases [44, 45]. On one hand, IFN-I response has been shown to have neuroprotective effects in viral infection of the CNS and multiple sclerosis [46]. On the other hand, excessive or prolonged activation of IFN-I response can lead to chronic inflammation and neurodegeneration. Microglial chimeras from patients with Down syndrome have an elevated expression of IFNARs compared to controls, whereas inhibiting the IFNARs expression increases the ramification of DS microglia and rescues their synaptic pruning functions [47]. In addition, amyloidosis leads to the progressive activation of microglia and a heightened microglia-mediated synaptic engulfment process in the brain by inducing the IFN-I responses [48]. In this study, miR-218-5p overexpression decreases the volume and aberrant phagocytosis in microglia of MPTP-injected mice, indicating that miR-218-5p restrains IFN-I responses. It has been reported that enhanced IFN-I responses in PD promote microglia-mediated neuroinflammation and neuron degeneration [49–52], whereas blocking IFN-I signaling rescues inflammation and the loss of DA neurons in various PD models [49, 53]. Consistent of these reports, we found miR-218-5p alleviated MPP^+^ induced inflammation by inhibiting IFN-I responses in microglia-like BV2 cells, suggesting that miR-218-5p may regulate neuroinflammation in PD through IFN-I signaling. Moreover, we identified miR-218-5p as a target gene of *Ddx41*, which has been implicated in IFN-I responses in previous studies [25, 26]. MiR-218-5p overexpression suppressed *Ddx41* expression in vivo and in vitro, despite the single binding site in the 3′UTR of *Ddx41*. *Ddx41* knockdown also partly reversed neuroinflammation in BV2 cells induced by MPP^+^-CM, suggesting that miR-218-5p regulates neuroinflammation by inhibiting *Ddx41* expression.

Our study has several limitations. First, we focused on the preventive role of miR-218-5p in our current work. However, it would be interesting to test if miR-218-5p can also reverse the damage caused by MPTP, as this would have important implications for therapeutic applications. In addition, IFN-I responses may occur in neurons with PD pathology and contribute to cell death [54]. Yet, whether miR-218-5p regulates neuron-mediated IFN-I responses remains unclear. Furthermore, the MPTP model does not induce α-synuclein accumulation [55], so this study did not examine the effect of the miR-218-5p-*Ddx41* axis on α-synuclein aggregation. Previous studies have demonstrated that α-synuclein aggregation also contributes to neurodegeneration and inflammation via the IFN-I pathway [51]. We hypothesize that miR-218-5p may exert its influence through this pathway. Moreover, the validation of miR-218-5p-*Ddx41* axis in PD requires clinical verification, and single miRNA therapy may cause severe adverse effects [56]. To ensure both safety and efficacy, further enhancements will be made regarding the in vivo drug delivery mode, dosage optimization, as well as exploring potential synergistic combinations with other miRNAs.

In conclusion, miR-218-5p alleviates microglia-mediated neuroinflammation and protects DA neurons from degeneration in PD by targeting *Ddx41* and regulating the IFN-I response. The miR-218-5p-*Ddx41* axis may represent a potential target for the treatment of PD.

### Supplementary Information

**Additional file 1: Table S1.** Primer sequences for quantitative real-time PCR. **Fig. S1.** Volcano plots showing the DEGs of RNA sequencing in miR-218 Control group versus NC Control group (**A**) and the DEGs in miR-218 MPTP group versus miR-218 Control group (**B**). N = 3 per group. Data are shown as |Log2 (fold change)| ≥ 0.585, *p*-value < 0.05. **Fig. S2.** Heatmaps of gene expression changes in the GO term “Response to interferon-beta” (**A**) and “Cellular response to interferon-beta” (**B**) based on RNA sequencing data from the SN of mice in the NC Control (NC PBS) group, NC MPTP group, miR-218 Control group and miR-218 MPTP group.

## Data Availability

The data that support the findings of this study are available from the corresponding author on reasonable request.

## Abbreviations

CNS: Central nervous system
CM: Conditioned media
DDX41: Dead-box helicase 41
DEGs: Differential expression genes
DA: Dopaminergic
DMEM: Dulbecco’s modified Eagle’s medium
FBS: Fetal bovine serum
FDR: False discovery rate
GO: Gene ontology
GSEA: Gene set enrichment analysis
MiR: MicroRNA
MPP^+^: 1-Methyl-4-phenylpyridinium
MPTP: 1-Methyl-4-phenyl-1,2,3,6-tetrahydropyridine
NES: Normalized enrichment score
PCA: Principal component analysis
PD: Parkinson’s disease
PBS: Phosphate buffer
PFA: Paraformaldehyde
qPCR: Quantitative real-time PCR
RNA-seq: RNA sequencing
SN: Substantia nigra
IFN-I: Type I interferon
WB: Western blot
3′UTR: 3′ Untranslated regions
6-OHDA: 6-Hydroxydopamine
