# Supplementary material for: MicroRNA-218-5p-Ddx41 axis restrains microglia-mediated neuroinflammation through downregulating type I interferon response in a mouse model of Parkinson’s disease
Source: J Transl Med. 2024 Jan 16;22:63. doi: 10.1186/s12967-024-04881-w (PMC10792813; doi:10.1186/s12967-024-04881-w)
Supplement: Supplementary file 1 — Additional file 1: Table S1. Primer sequences for quantitative real-time PCR. Fig. S1. Volcano plots showing the DEGs of RNA sequencing in miR-218 Control group versus NC Control group (A) and the DEGs in miR-218 MPTP group versus miR-218 Control group (B). N = 3 per group. Data are shown as |Log2 (fold change)| ≥ 0.585, p-value < 0.05. Fig. S2. Heatmaps of gene expression changes in the GO term “Response to interferon-beta” (A) and “Cellular response to interferon-beta” (B) based on RNA sequencing data from the SN of mice in the NC Control (NC PBS) group, NC MPTP group, miR-218 Control group and miR-218 MPTP group. [file 12967_2024_4881_MOESM1_ESM.docx]

**Table S1 Primer sequences for quantitative real-time PCR**

| **Gene** | **Forward (5’-3’)** | **Reverse (5’-3’)** |
| --- | --- | --- |
| Actb | GTACTCTGTGTGGATCGGTGG | AAAACGCAGCTCAGTAACAGTC |
| Irf7 | CCGTGTTTACGAGGAACCCT | TACAGGAACACGCATCTGGG |
| Nlrc5 | TTGCCTACTATGGGGAGCCT | AGCTCCACAAGACTCAGCAC |
| Ddx60 | AAGTGATGAGCCTTTGTTGAGG | CTCCCACATTCAAATCCAGGC |
| Ifnb1 | CGTGGGAGATGTCCTCAACT | AGATCTCTGCTCGGACCACC |
| Il1b | TGCCACCTTTTGACAGTGATG | AAGGTCCACGGGAAAGACAC |
| Il6 | TCTTTGAAGTTGACGGACCC | TGAGTGATACTGCCTGCCTG |
| Tnf | AGACCCTCACACTCACAAACCAC | GCACGTAGTCGGGGCAGC |


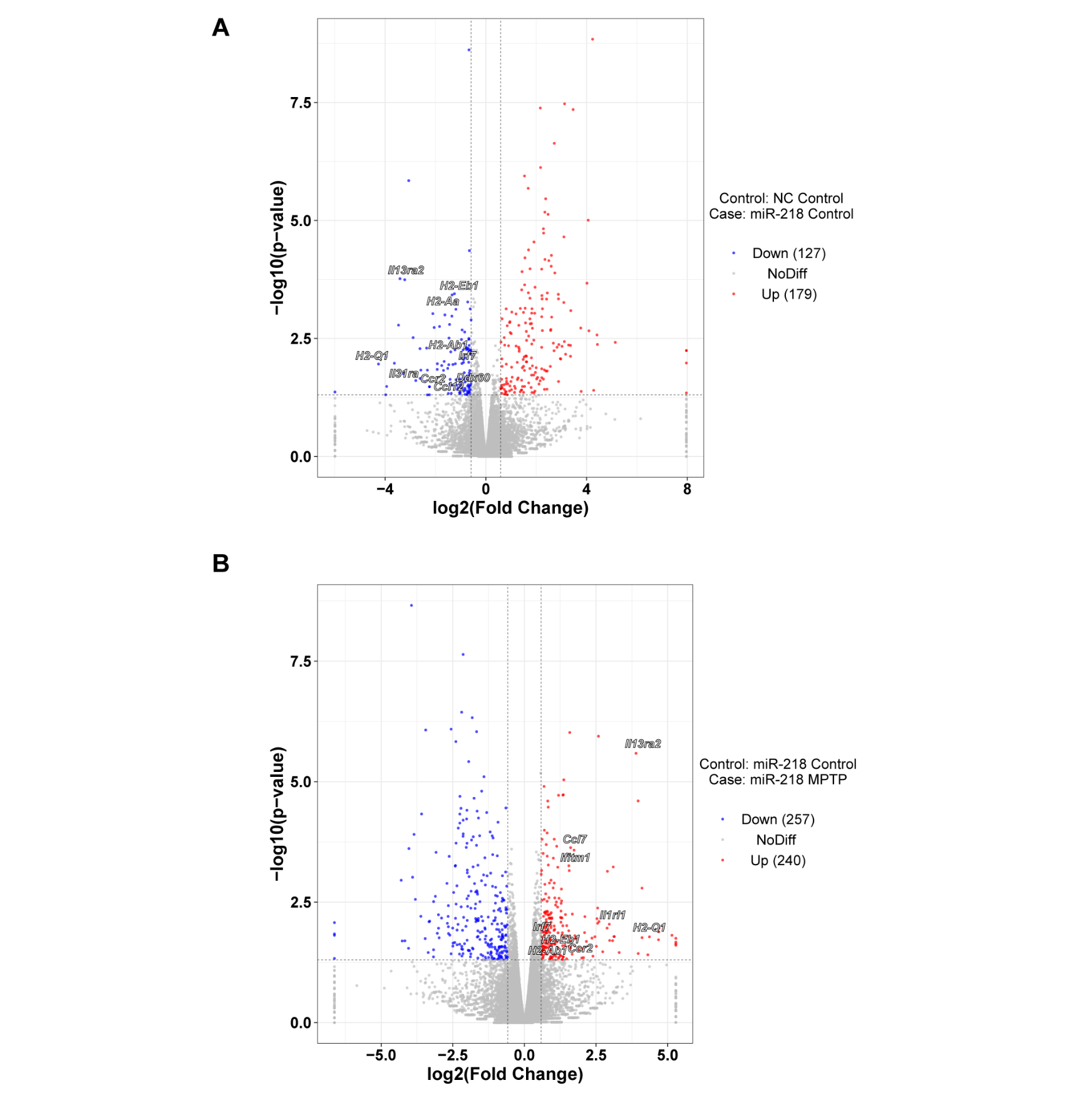


**Figure S1** Volcano plots showing the DEGs of RNA sequencing in miR-218 Control group vs. NC Control group **(A)** and the DEGs in miR-218 MPTP group vs. miR-218 Control group **(B)**. N=3 per group. Data are shown as |Log2 (fold change)|≥ 0.585, p-value < 0.05.


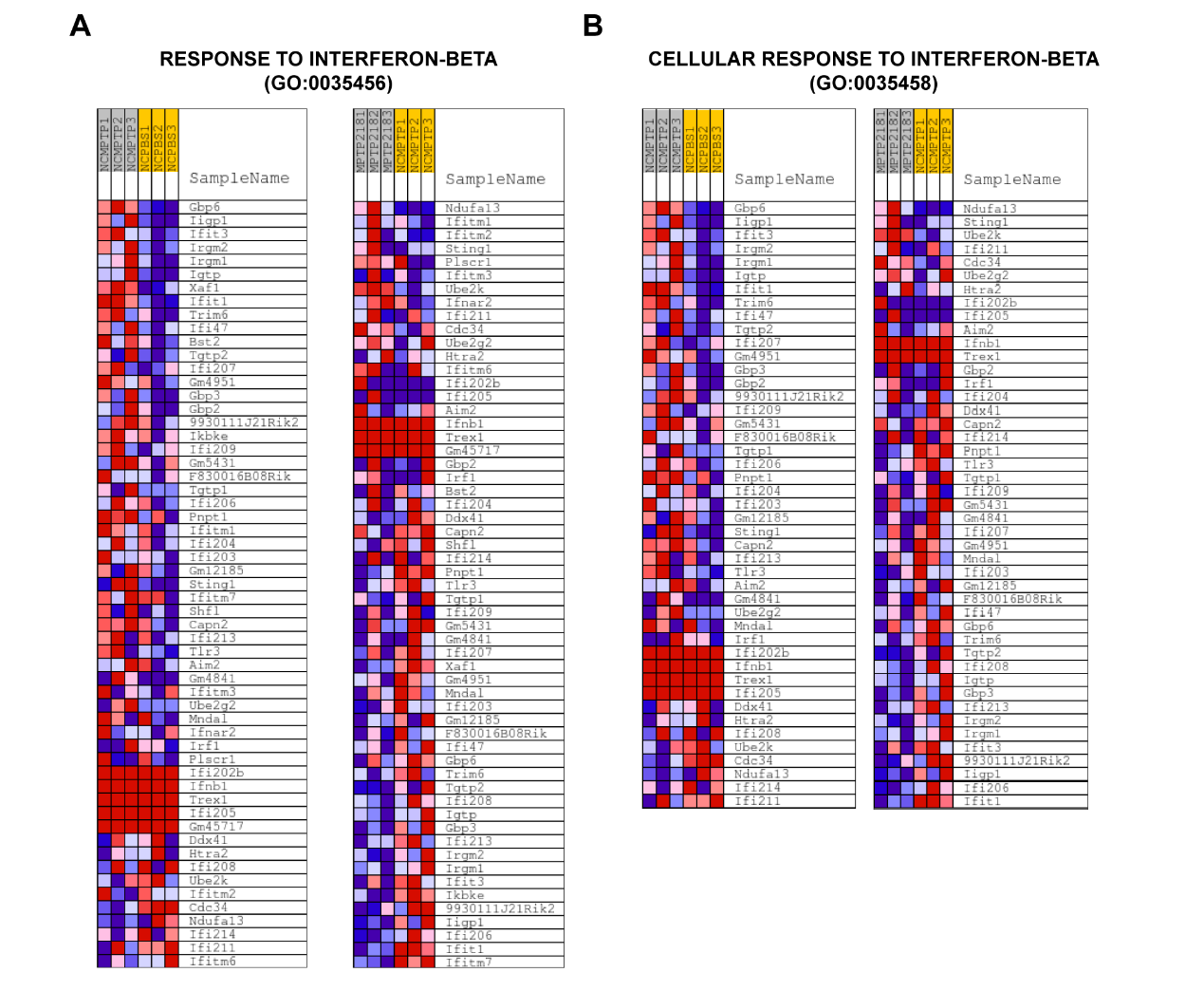


**Figure S2** Heatmaps of gene expression changes in the GO term "Response to interferon-beta" **(A)** and "Cellular response to interferon-beta" **(B)** based on RNA sequencing data from the SN of mice in the NC Control (NC PBS) group, NC MPTP group, miR-218 Control group and miR-218 MPTP group.
